# Therapeutic Potential of Targeting the Oxytocinergic System

**DOI:** 10.3390/ijms232113295

**Published:** 2022-10-31

**Authors:** Adele Romano, Gustavo Provensi

**Affiliations:** 1 Department of Physiology and Pharmacology “V. Erspamer”, Sapienza University of Rome, 00185 Rome, Italy; 2 Department of Neuroscience, Psychology, Drug Research and Child Health (NEUROFARBA), Section of Pharmacology of Toxicology, University of Florence, 50139 Florence, Italy

The nine amino acid neuropeptide oxytocin (OXT, Cys–Tyr–Ile–Gln–Asn–Cys–Pro–Leu–Gly–NH2) is one of the most studied hormones of the body. It is mainly synthesized at the paraventricular (PVN) and supraoptic (SON) nuclei of the hypothalamus. Within these brain regions, two cellular types are responsible for oxytocin production: the magnocellular and the parvocellular cells. The magnocellular neurons are the principal source of oxytocin production. These cells mainly project to the neurohypophysis, from where oxytocin is released into the bloodstream. Parvocellular neurons, instead, innervate distinct areas of the brainstem, midbrain, and the spinal cord [1]. In addition, many studies have shown that oxytocin neurons may influence the central nervous system through dendritic and somatic release [2]. To date, only one specific OXT receptor (OXTR) was described. This receptor belongs to the class-A/rhodopsin of the G protein-coupled receptor family. Indeed, OXTRs are coupled to Gq/11, a class of GTP binding proteins that stimulate the phospholipase C signaling pathway. OXTRs are expressed in several tissues in the body: in periphery, they are found in the heart, kidney, thymus, adipocyte tissue, gastrointestinal tract, mammary glands, and uterus; at central level they are widely distributed being densely expressed in the nucleus accumbens (NAc), prefrontal cortex (PFC), anterior olfactory nucleus, lateral septum (LS), bed nucleus of the stria terminalis (BNST), amygdala, and hippocampus [3].

Based on the vast distribution of OXTR in the body at both central and peripheral level, it is not surprising that oxytocin modulates a wide range of neurotransmitter and neuromodulator activities as well as physiologic functions and behaviors, including social interactions, social memory response to social stimuli, decision making, feeding behavior, emotional reactivity, aggressiveness, maternal behavior, sexual behavior, and so on. Thus, the oxytocinergic system continues to be the focus of intense exploratory research in animals and humans, as a possible therapeutic agent for the treatment of different neuropsychiatric disorders such as autism, eating disorders, schizophrenia, and anxiety-related disorders. The potential use of oxytocin in these disorders is attracting growing interest since numerous beneficial properties are ascribed to this neuropeptide (Figure 1 and Table 1). In this scenario, the aim of this Special Issue was to collect evidence about OXT as a research tool and therapeutic agent to highlight the need for further investigations that might contribute to the development of novel, safe, and successful therapies.

In this scenario, Friuli et al. [4], reported evidence demonstrating the potential implication of OXT in the modulation of inflammation occurring in different chronic diseases, including neurological, gastrointestinal and cardiovascular disorders, diabetes and obesity. The evidence reviewed supports a beneficial role of OXT in the control of both peripheral and central inflammatory response happening in the aforementioned pathologies. Although future studies are necessary to elucidate the mechanistic details underlying such regulation, this study supports the idea that the modulation of the endogenous oxytocinergic system might represent a new potential pharmacological approach for the treatment of inflammation.

Several studies reported sexual dimorphisms in terms of oxytocin levels, OXTR expression and oxytocin-induced responses. The work of Wang et al. [5], further supports these sex differences by showing that the chemogenetic activation of PVN oxytocin neurons increased attention towards novel objects in male, but not in female rats. Using an optogenetic approach, these authors demonstrated that OXT neurons in the PNV OXT fibers excite noradrenergic neurons in the locus coeruleus, a brain region essential for cognitive focus and attention, by co-release of OXT and glutamate. Interestingly also the magnitude of the photoactivated excitatory neurotransmission was greater in males than females. The larger synaptic response in males is unlikely due to differences in postsynaptic glutamate receptor density, or the amount of OXT released in this neurotransmission, but rather may be due to increased glutamate release in males compared to females.

There is a clear increase in substance-abuse disorders and drug overdose deaths in recent years. Factors related to the COVID pandemic, such as social isolation, stress, and decreased access to substance use disorder treatment and emergency services made the problem worse. In this scenario, there is a clear need for identifying new effective pharmacotherapeutics for addiction. A vast body of literature highlights the reciprocal relationship between OXT and drugs of abuse: the OXT system can be altered by acute or chronic exposure to drugs of abuse and vice versa OXT can modulate the individual’s response to the drugs. In this regard, the reviews of Ferrer-Perez et al. [6] and Sundar et al. [7] deal with a very interesting modulatory function exerted by OXT on reward-related and addiction-related neural substrates and mechanisms. Ferrer-Perez et al. [6] highlights the role of the role played by OXT in modulating vulnerability or resilience to developing a substance use disorder, placing specific attention on the role of social stress as a risk factor of addiction. The studies summarized in this review strongly demonstrate the therapeutic potential of exogenous OXT in modulating the initial response to drugs of abuse, in attenuating of the development of dependence, and blunting drug reinstatement. In Sundar et al. [7], the potential mechanisms through which OXT attenuates reward-seeking behaviors and the reinstatement for drugs of abuse and natural rewards are discussed. Particular attention is devoted to the interactions between OXT impacts on glutamatergic, dopaminergic and GABAergic neurotransmission systems. 

Perinatal maternal stress (during pregnancy and neonatal stages) is a major risk predisposing the offspring to negative outcomes, including compromised cognitive, social-emotional, and neural development. To mimic a strong stress axis activation during the perinatal period, Laviola et al. [8], exposed female mouse dams to abnormal levels of corticosterone during the last week of pregnancy or the first one of lactation and evaluated the long-term impacts of such manipulation in the offspring during adulthood. Mice perinatal exposed to corticosterone presented exaggerated response to stress, either in social and emotional domains as revealed by aberrant persistence of adolescence-typical increased interest towards novel social stimuli and deficient emotional contagion. An increased expression of OXTR was found in the hypothalamus as well as in the cortex of postnatally corticosterone-exposed mice. Intranasal oxytocin administration, reduced stress responses and restored a regular behavioral phenotype.

Emotional experiences leave long-lasting traces in the brain. Indeed, the persistence of the memory related to aversive events is fundamental for individuals to adapt their behavior in order to respond adequately to treat situations. Disruption of one or more elements of mnemonic processing may result in intrusive memories, triggering maladaptive responses that can form the basis of various psychiatric disorders, including generalized anxiety, obsessive compulsive disorders, PTSD and specific phobias. Currently, the gold standard treatment for these disorders is the exposure-based therapy, a behavioural cognitive therapy conceptually based upon fear extinction. One strategy to overcome some limits and improve the efficacy of the exposure therapy is the combination with pharmacological agents. In this regard, the review of Baldi et al. [9] aims to answer the following question: is there a role for OXT as an adjunctive therapy for the treatment of fear disorders? In this paper, the authors provide an extensive overview of the complex networks underpinning fear memory extinction and critically discuss the recent literature addressing the contradictory effects of OXT on fear extinction at preclinical and clinical levels.

Finally, although future studies are needed, the evidence collected in this Special Issue strongly supports the idea that the oxytocinergic system might represent one of the mechanisms that participate in the coordination of different aspects of a variety of diseases spanning in different domains, becoming an interesting pharmacological tool for the development of novel therapies.

## Figures and Tables

**Figure 1:**
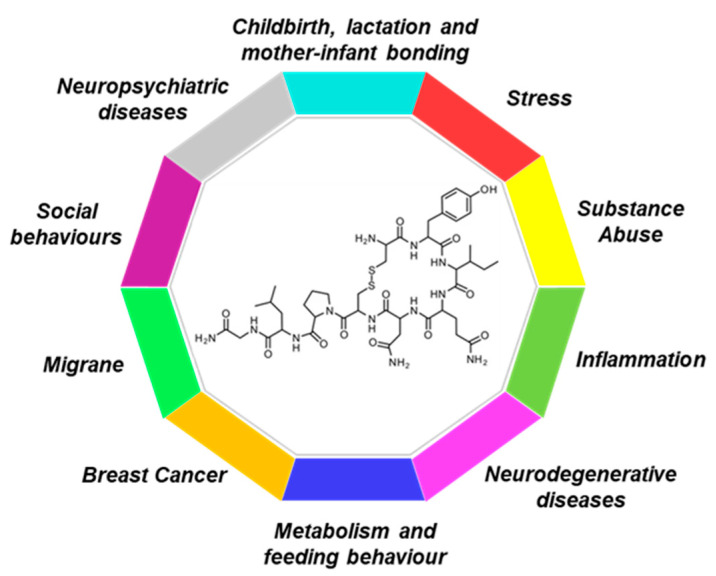
Therapeutic Potential of Targeting the Oxytocinergic System.

**Table 1:** Preclinical and clinical evidence of oxytocin potentiality in several physiological and pathological conditions.

Condition	Sientific Evidence	Preclinical	Clinical
Breast Cancer	Potential protective role in breast cancer	X	X
Childbirth, lactation and mother-infant bonding	Induction of labour		X
	Involution of the post-partum uterus through tonal contractility of the uterine muscles	X	X
	Stimulation of milk release from the mammary glands through the lactiferous ducts	X	X
	Facilitation of maternal care behaviors	X	X
	Potential role in preventing mother’s PTSD following childbirth	X	X
Inflammation	Alleviation of oxidative stress and inflammation murine model of ASD	X	
	Improvement of inflammation on ovarian tumor cells and after I/R injury	X	
	Control of peripheral and central inflammatory response occurring in chronic diseases	X	X
Metabolism and feeding behaviour	Increase in energy expenditure and lipolysis and improvement of glucose homeostasis	X	
	Decrease in the intake of palatable and sweet foods	X	
	Anabolic effect on lean mass and bone mineral density	X	
	Reduction in caloric intake, increase in fat oxidation, improvement of insulin sensitivity	X	X
	Modulation of both homeostatic and reward-driven food intake	X	X
Migraine	Reduction in pain, decrease in the frequency of headaches in both chronic and high frequency episodic migraineurs		X
Neuropsychiatric diseases	Improvement of the symptoms associated with depression (sleep disorders and anhedonia)	X	
	Increase in social reward and working memory in PTSD patients		X
	Improvement of the social function of autistic patients (processing of social information, empathy and social communication ability)		X
	Amelioration of both the positive and negative symptoms and restoration of social cognitive deficits in patients with schizophrenia		X
	Anxiolytic and antidepressant effects	X	X
Neurodegenerative diseases	Neuroprotective potential in Alzheimer’s disease by restoring cognition and suppressing β-amyloid, Tau accumulation, and neuronal death	X	
	Amelioration of the locomotor disabilities and anxiety-like behaviors and improvement of oxidative stress in MPTP mice	X	
Social behaviours	Decrease hyper aggressive behaviour, accompanied by an increase in social contact	X	
	Potential inhibition of aggression and violent behavior		X
	Pro-social effect, Modulation of fear mmemory extinction	X	X
Substance Abuse	Decrease in locomotor hyperactivity and stereotyped behaviours after exposure to psychostimulants such as cocaine and methamphetamine	X	
	Anticraving properties in drug addiction (including alcohol and opioids)	X	X
	Promoting of positive social interactions and social reward in drug addiction	X	X
Stress	Promotion of neuronal regeneration processes, rescuing the suppression of neurogenesis processes induced by prolonged exposure to stress episodes and glucocorticoids;	X	
	Modulation of stress responses and facilitation of adaptation to stress	X	X
